# A Case of Pneumonia with Cerebral Complications

**Published:** 1898-02-26

**Authors:** Sidney Coupland

**Affiliations:** Physician to the Hospital


### Medical Progress and Hospital Clinics.

[*The Editor will be glad to receive offers of co-operation and contributions from members of the profession. All letters should be addressed to* The Editor, at the Office, 28 & 29, Southampton Street, Strand, London, W.C.]

### A CASE OF PNEUMONIA WITH CEREBRAL COMPLICATIONS.

A Clinical Lecture given at the Middlesex Hospital by Sidney Coupland, M.D., F.R.C.P., Physician to the Hospital.

(*Concluded from page* 359.)

I do not propose to enter into any discussion of the nature of delirium and its relations with insanity. It would take me too far, as well as too long, and as the whole subject is more or less conjectural, it would be of doubtful profit, either to you or to myself. But I should like to point out, and to impress the fact strongly, that pneumonia is not unfrequently complicated with cerebral disturbance of a more definite, and, as regards life, a more dangerous character than even delirium or mania. In some cases acute meningitis develops, and the "pneumococcus" has been found in the inflammatory exudation. In others a condition which must, for want of a better term, be called "cerebritis," may occur, and that, too, quite early in the disease, and be fatal even before any pulmonary change has been defined. This may seem strange beyond belief, to have an individual dying from pneumonia before the lungs are inflamed; and yet I think there is a real basis for it. So far as we know pneumonia requires for its development the presence of a special agent—the microbe "pneumococcus" and its toxine—and from the varied "complications" of acute pneumonia we are aware of this same agent affecting similarly other organs. If this specific poison were to act primarily, and with sufficient intensity on the brain, might it not early give rise to grave cerebral symptoms? This idea has grown up in my own mind as the result of some recent experiences. About two years ago I was asked by the Home Office to inquire into the cause and nature of some cases of mysterious and suddenly fatal illness which had occurred from time to time amongst the inmates of a reformatory school for boys n the North of England. Within the space of nine years there had been in all nine such deaths, of boys of varying age and occupation, all, of course, of the lowest class of life, but most without any antecedent history of ill-health. Their illnesses occurred with remarkable suddenness, and were of a cerebral type, characterised by vomiting, high temperature, headache, stupor, and ending mostly, within a few hours—twelve to twenty-four—in death by coma. In one or two instances inquests had been held, and in the cases that were seen during life by a medical man the cause of death was certified as "congestion of the brain" or some equally vague condition. I satisfied myself that there was nothing special to the mode of life or surroundings to account for these cases, but I was at a loss to arrive at a definite conclusion. To be sure, I did see one patient suffering from pneumonia and pericarditis whose illness had begun with somewhat similar symptoms to those stated to have characterised the fatal cases. I might never have arrived at a conclusion had it not been that, singularly enough, about the time of my visit one or two similar fatal illnesses had occurred in a girls' industrial school in the same neighbourhood. In one of these a post-mortem examination was made by a well-known pathologist, who found nothing but extreme congestion of internal organs, such as might, he thought, characterise the onset of some specific fever. A year passed, when another case occurred in this latter school, and, in accordance with my wish, I was sent for to make a post-mortem examination. The girl, apparently well the day before, had complained of headache, vomited, was sent to bed, and found dead in the morning. The post-mortem revealed vascularity of brain, swelling of spleen, and an area of recent pneumonic consolidation covered by flakes of pleuritic lymph in one lung. Nor was this all; for an almost analogous case, except that it was briefer in duration, occurred a few months later, and I again found a pneumonic focus, of which the microscopic slides are before you. I am convinced that these two cases, and I infer that others which resembled them clinically, were due to the same virus that excited the pneumonia acting with especial intensity upon the brain; and my object in mentioning them now in connection with the case of the patient in Hertford ward is to emphasise the fact that the pneumonic virus is one which may at times act as a cerebral poison, and that to such an extent as to produce something more than excitation of delirium, but even coma and death.

The records of pneumonia contain cases almost as striking where the fact of pulmonary inflammation has been lost sight of in the predominance of violent cerebral disturbance, cases which have been styled sometimes "cerebral" pneumonia, sometimes "latent" pneumonia, but where the end has come suddenly and from cerebral or nervous disorder rather than from pulmonary. It is the existence of such cases that force us to take a wide view of the nature of this remarkable affection, just as its whole course and general symptomatology impress the fact that it is something more than a local disease. You will often find that there is more general disturbance, more delirium, more danger in a case that presents locally the signs of involvement of a small area (*e g*, the apex of the lung) than one where a large tract of lung is affected.

The conclusion of the whole matter is therefore this: Acute lobar pneumonia is a specific febrile disease due to a specific cause, generally (perhaps not invariably) the pneumococcus; that this agent may, and often does, exert a baneful influence upon other organs, initiating in them inflammatory changes analogous to those in the lung, or, if the brain be the organ it especially attacks, actually causing death before any distinctive inflammatory changes are set up; and that it is probable that the delirium and other nervous symptoms of pneumonia (apart from the alcoholic delirium tremens) are mainly due to the direct toxic effect of this same agent.

